# Supplementary material for: Post-translational thioamidation of methyl-coenzyme M reductase, a key enzyme in methanogenic and methanotrophic Archaea
Source: eLife. 2017 Sep 7;6:e29218. doi: 10.7554/eLife.29218 (PMC5589413; doi:10.7554/eLife.29218)
Supplement: Supplementary file 1. [file elife-29218-supp1.docx]

**Supplementary File 1:** List of plasmids used in this study

| Plasmid | Features | Source |
| --- | --- | --- |
| pAMG40 | Vector for fosmid retrofitting that contains pC2A and λattB | (Guss et al. 2008) |
| pJK027A | Vector with P*mcrB*(*tetO1*) promoter fusion to *uidA* that contains φC31-attB and λattP | (Guss et al. 2008) |
| pJK029A | Vector with P*mcrB*(*tetO4*) promoter fusion to *uidA* that contains φC31-attB and λattP | (Guss et al. 2008) |
| pDN201 | pJK027A-derived plasmid with P*mcrB*(*tetO1*) promoter fusion to Spy *cas9* | (Nayak and Metcalf 2017) |
| pDN244 | pDN201-derived plasmid with synthetic fragments containing P*mtaCB1* promoter fusion to sgRNAs in tandem targeting the *ycaO-tfuA* coding sequences(CDS) | This study |
| pDN245 | pDN244-derived plasmid containing a repair template to generate an in-frame deletion of the *ycaO-tfuA* CDS | This study |
| pDN246 | Cointegrate of pDN244 and pAMG40 | This study |
| pDN247 | Cointegrate of pDN245 and pAMG40 | This study |
| pDN258 | pDN201-derived plasmid with synthetic fragments containing P*mtaCB1* promoter fusion to sgRNAs in tandem targeting the *mcrA* CDS | (Nayak and Metcalf 2017) |
| pDN259 | pDN258-derived plasmid containing a repair template to generate an in-frame deletion of the *mcrA* CDS | (Nayak and Metcalf 2017) |
| pDN260 | Cointegrate of pDN258 and pAMG40 | (Nayak and Metcalf 2017) |
| pDN261 | Cointegrate of pDN259 and pAMG40 | (Nayak and Metcalf 2017) |
| pDN303 | pDN201-derived plasmid with synthetic fragments containing P*mtaCB1* promoter fusion to a sgRNA targeting *mcrG* | This study |
| pDN305 | pDN303-derived plasmid containing a repair template to introduce a tandem affinity purification tag (containing a 3X FLAG tag and a Twin-Strep tag) at the N-terminus of *mcrG* | This study |
| pDN307 | Cointegrate of pDN303 and pAMG40 | This study |
| pDN309 | Cointegrate of pDN305 and pAMG40 | This study |
| pDN314 | pDN201-derived plasmid with synthetic fragments containing P*mtaCB1* promoter fusion to sgRNAs in tandem targeting the *ycaO* CDS | This study |
| pDN315 | pDN314-derived plasmid containing a repair template to generate an in-frame deletion of the *ycaO* CDS | This study |
| pDN316 | Cointegrate of pDN314 and pAMG40 | This study |
| pDN317 | Cointegrate of pDN315 and pAMG40 | This study |
| pDN318 | pDN201-derived plasmid with synthetic fragments containing P*mtaCB1* promoter fusion to sgRNAs in tandem targeting the *tfuA* CDS | This study |
| pDN319 | pDN318-derived plasmid containing a repair template to generate an in-frame deletion of the *tfuA* CDS | This study |
| pDN320 | Cointegrate of pDN318 and pAMG40 | This study |
| pDN321 | Cointegrate of pDN319 and pAMG40 | This study |
| pDN342 | pJK029A-derived plasmid with P*mcrB*(*tetO4*) promoter fusion to *ycaO-tfuA* CDS from *M. acetivorans* | This study |
| pDN343 | pJK029A-derived plasmid with P*mcrB*(*tetO4*) promoter fusion to *ycaO*  CDS from *M. acetivorans* | This study |
| pDN344 | pJK029A-derived plasmid with P*mcrB*(*tetO4*) promoter fusion to *tfuA* CDS from *M. acetivorans* | This study |
| pDN345 | Cointegrate of pDN342 and pAMG40 | This study |
| pDN346 | Cointegrate of pDN343 and pAMG40 | This study |
| pDN347 | Cointegrate of pDN344 and pAMG40 | This study |
