# Supplementary material for: Post-translational thioamidation of methyl-coenzyme M reductase, a key enzyme in methanogenic and methanotrophic Archaea
Source: eLife. 2017 Sep 7;6:e29218. doi: 10.7554/eLife.29218 (PMC5589413; doi:10.7554/eLife.29218)
Supplement: Supplementary file 2. [file elife-29218-supp2.docx]

**Supplementary File 2:** List of target sequences used in this study

| Gene (locus tag) | Target sequence (+ PAM) | Position on *M. acetivorans* chromosome |
| --- | --- | --- |
| *ycaO* (MA0165) | GGC TTC GTC ATA CAC GCG CTG GG | 196357-196376 (+ strand) |
| *tfuA* (MA0164) | TTG TAA GCC CGA GAT AGC TCC GG | 194638-194657 (+ strand) |
| *mcrA* (MA4546) | TGA ACT CTC TGA TGG CAC CG CGG | 5596716-5596735 (+ strand) |
| *mcrA* (MA4546) | GAT TGC ACG CTG ACC GAG AG GGG | 5598139-5598158 (+ strand) |
| *mcrG*  (MA4547) | CTT TCT TCT GTT AGC GCC GA CGG | 5599085-5599107 (+ strand) |
| *ycaO_*1  (MA0165) | GCT CGG GAT TCC GAT CTT CT CGG | 196257-196279 (- strand) |
| *ycaO_*2  (MA0165) | CAG TGA TTC TTT GTC CTT TG CGG | 195210-195232 (+ strand) |
| *tfuA_*1  (MA0164) | GGG GCT GGT AGT TTG CCC TG AGG | 195070-195092 (- strand) |
| *tfuA_*2  (MA0164) | TTA TAT CAA CTT CGC TGT TA AGG | 194554-194576 (+ strand) |
