# Supplementary material for: Post-translational thioamidation of methyl-coenzyme M reductase, a key enzyme in methanogenic and methanotrophic Archaea
Source: eLife. 2017 Sep 7;6:e29218. doi: 10.7554/eLife.29218 (PMC5589413; doi:10.7554/eLife.29218)
Supplement: Supplementary file 3. [file elife-29218-supp3.docx]

**Supplementary File 3:** List of *Methanosarcina acetivorans* strains used in this study

| Strain | Genotype | Construction details | Source |
| --- | --- | --- | --- |
| WWM60 | Δ*hpt*::*PmcrB-tetR* | --- | (Guss et al. 2008) |
| WWM992 | Δ*hpt*::*PmcrB-tetR, ΔycaO-tfuA* | WWM60 was transformed to Pur^R^ with pDN247; plasmid-cured strain was isolated by colony purifying Pur^R^ transformants on solid medium with 8ADP | This study |
| WWM1054 | Δ*hpt*::*PmcrB-tetR,* N-terminal TAP tag (3X FLAG and Twin-Strep tag) upstream of *mcrG* | WWM60 was transformed to Pur^R^ with pDN309; plasmid-cured strain was isolated by colony purifying Pur^R^ transformants on solid medium with 8ADP | This study |
| WWM1064 | Δ*hpt*::*PmcrB-tetR, ΔycaO* | WWM60 was transformed to Pur^R^ with pDN317; plasmid-cured strain was isolated by colony purifying Pur^R^ transformants on solid medium with 8ADP | This study |
| WWM1066 | Δ*hpt*::*PmcrB-tetR, ΔtfuA* | WWM60 was transformed to Pur^R^ with pDN321; plasmid-cured strain was isolated by colony purifying Pur^R^ transformants on solid medium with 8ADP | This study |
| WWM1076 | Δ*hpt*::*PmcrB-tetR, ΔycaO-tfuA,* N-terminal TAP tag (3X FLAG and Twin-Strep tag) upstream of *mcrG* | WWM1054 was transformed to Pur^R^ with pDN247; plasmid-cured strain was isolated by colony purifying Pur^R^ transformants on solid medium with 8ADP | This study |
| WWM1087 | Δ*hpt*::*PmcrB-tetR, ΔycaO-tfuA/* pDN345 [*PmcrB(tetO4)-ycaO-tfuA*] | WWM992 was transformed to Pur^R^ with pDN345; isolates were verified by Sanger sequencing and grown in HS medium with 2 µg/mL Puromycin | This study |
| WWM1088 | Δ*hpt*::*PmcrB-tetR, ΔycaO-tfuA/* pDN346 [*PmcrB(tetO4)-ycaO*] | WWM1064 was transformed to Pur^R^ with pDN346; isolates were verified by Sanger sequencing and grown in HS medium with 2 µg/mL Puromycin | This study |
| WWM1089 | Δ*hpt*::*PmcrB-tetR, ΔycaO-tfuA/* pDN347 [*PmcrB(tetO4)-tfuA*] | WWM1066 was transformed to Pur^R^ with pDN347; isolates were verified by Sanger sequencing and grown in HS medium with 2 µg/mL Puromycin | This study |
